# Impaired skeletal muscle performance as a consequence of random functional capillary rarefaction can be restored with overload‐dependent angiogenesis

**DOI:** 10.1113/JP278975

**Published:** 2020-02-26

**Authors:** Peter G Tickle, Paul W Hendrickse, Hans Degens, Stuart Egginton

**Affiliations:** ^1^ School of Biomedical Sciences University of Leeds UK; ^2^ Department of Life Sciences Manchester Metropolitan University UK; ^3^ Institute of Sport Science and Innovations Lithuanian Sports University Lithuania

**Keywords:** blood flow, fatigue, ischaemia, microcirculation, microspheres, skeletal muscle

## Abstract

**Key points:**

- Loss of skeletal muscle capillaries is thought to contribute to a reduction in exercise tolerance, but the relative contribution of a compromised microcirculation with disease, in isolation of co‐morbidities, to impaired muscle function is unknown.
- We therefore developed a novel method to randomly occlude capillaries in the rat hindlimb to mimic the capillary rarefaction observed in many conditions.
- We demonstrate that muscle fatigue resistance is closely coupled with functional microvascular density, independent of arterial blood flow, while disturbance of the microcirculation leads to long‐term impairment of muscle function if left untreated.
- Mechanical stretch due to muscle overload causes a restoration of fatigue resistance via angiogenic remodelling.
- These observations highlight the importance of a healthy microcirculation and suggest that restoring impaired microvascular supply, regardless of disease co‐morbidities, will assist recovery of exercise tolerance in a variety of conditions that limit quality of life.

**Abstract:**

To what extent microvascular rarefaction contributes to impaired skeletal muscle function remains unknown. Our understanding of whether pathological changes in the microcirculation can be reversed remains limited by a lack of basic physiological data in otherwise healthy tissue. The principal objectives here were to: (1) quantify the effect of random microvascular rarefaction on limb perfusion and muscle performance, and (2) determine if these changes could be reversed. We developed a novel protocol in rats whereby microspheres injected into the femoral artery allowed a unilateral reduction in functional capillary density in the extensor digitorum longus (EDL), and assessed acute and chronic effects on muscle function. Simultaneous bilateral EDL force and hindlimb blood flow measurements were made during electrical stimulation. Following functional capillary rarefaction there was an acute microsphere dose‐dependent reduction in muscle fatigue resistance (*P *< 0.001), despite preserved femoral artery perfusion. Histological analysis of EDL samples taken from injected animals confirmed a positive correlation between the proportion of functional capillaries and fatigue resistance (*P* = 0.002). Such impaired performance persisted for at least 2 weeks (*P* = 0.016). Concomitant mechanical overload improved both perfused capillary density and fatigue resistance (*P*<0.05), confirming that the capacity for muscle remodelling was retained following chronic distributed ischaemia, and that the impact of capillary rarefaction could be alleviated. These results demonstrate that loss of functional capillaries is detrimental to muscle function, even in otherwise healthy tissue, independent of arterial perfusion. Restoration of muscle performance following a mechanical overload stimulus indicates that angiogenic treatments to alleviate microvascular rarefaction may be key to restoring exercise tolerance.

## Key points

- Loss of skeletal muscle capillaries is thought to contribute to a reduction in exercise tolerance, but the relative contribution of a compromised microcirculation with disease, in isolation of co‐morbidities, to impaired muscle function is unknown.
- We therefore developed a novel method to randomly occlude capillaries in the rat hindlimb to mimic the capillary rarefaction observed in many conditions.
- We demonstrate that muscle fatigue resistance is closely coupled with functional microvascular density, independent of arterial blood flow, while disturbance of the microcirculation leads to long‐term impairment of muscle function if left untreated.
- Mechanical stretch due to muscle overload causes a restoration of fatigue resistance via angiogenic remodelling.
- These observations highlight the importance of a healthy microcirculation and suggest that restoring impaired microvascular supply, regardless of disease co‐morbidities, will assist recovery of exercise tolerance in a variety of conditions that limit quality of life.

## Introduction

Critical functions of the microcirculation that optimise muscle performance during exercise include supporting adequate gas exchange, delivery of nutrients and removal of metabolites. The extent of microvascular supply in skeletal muscle is associated with the integrative effects of activity (Andersen & Henriksson, 1977; Jensen *et al*. 2004; Hoier *et al*. 2012; Gavin *et al*. 2015) in a feedback manner, such that muscles with experimentally increased capillary density exhibit increased fatigue resistance (Hudlická *et al*. 1977). In contrast, reduced capillarity occurs in skeletal muscle after experimentally induced (Kindig *et al*. 1999; Nusz *et al*. 2003) and clinical (Schaufelberger *et al*. 1995; Duscha *et al*. 1999; Wadowski *et al*. 2018) chronic heart failure (CHF), from which we can infer that poor fatigue resistance in disease may be accounted for, at least in part, by deleterious changes in the microcirculation.

Not only the number but also the distribution of capillaries plays an important role in tissue oxygenation (Degens *et al*. 1994; Egginton & Gaffney, 2010). If capillary loss is also accompanied by an increased heterogeneity of capillary spacing in the muscle tissue, then this may cause tissue oxygenation to be reduced more than expected from reduced capillary density alone (Piiper & Scheid, 1991; Degens *et al*. 2006), and hence contribute to a further decline in functional capacity of the muscle.

Impaired oxygen supply in disease may also be exacerbated by a reduction in the proportion of capillaries supporting continuous red blood cell flow (Kindig *et al*. 1999; Richardson *et al*. 2003). In support of this, an ∼4‐fold reduction in mechanical output of the heart resulted from a stochastic occlusion of 25% of terminal arterioles in the coronary microcirculation, suggesting that distributive (microvascular) ischaemia may be more detrimental than that with a focal (arterial) origin (Hauton *et al*. 2015). Thus, microvascular rarefaction *per se* may represent a mechanism for exercise intolerance associated with diseases adversely affecting the microcirculation (e.g. CHF, diabetes). A lack of experimental data on the physiological effects of microvascular rarefaction, in the absence of influence from concomitant changes due to disease, presents a constraint to our understanding of the interaction between muscle performance and capillary supply. Consequently, it is imperative that the basic physiological effects that arise from a compromised microcirculation are established. The primary objective of this study was therefore to assess the effect on muscle performance of an isolated reduction in functional microvascular density, in otherwise healthy tissue.

Future benefits of deciphering this relationship may include modifying existing treatments (Zamani *et al*. 2017) to recover impaired exercise tolerance in microvascular disease. We therefore studied how muscle function and blood flow were affected by acute and chronic reductions in the number of functional (perfused) capillaries, and how the angiogenic response to mechanical overload (Degens *et al*. 1992; Zhou *et al*. 1998; Deveci & Egginton, 2002; Egginton *et al*. 2011; Ballak *et al*. 2016) was affected by an underlying constrained microcirculation in otherwise healthy tissue. Compensatory overload after synergist extirpation occurs due to the additional functional demands imposed on remaining muscles. Pronounced hypertrophy (Zhou *et al*. 1998; Deveci & Egginton, 2002) coupled with increased fatigue resistance (Frischknecht & Vrbova, 1991; Ballak *et al*. 2016) due to angiogenic remodelling (Egginton *et al*. 2011, 1998; Williams *et al*. 2006; Ballak *et al*. 2016) proceeds shortly after overload surgery.

Specifically, we tested the hypotheses that fatigue resistance will be reduced in proportion to the decreased number of functional capillaries, but that the capacity for remodelling is retained such that the angiogenic mechanical stimulus of muscle overload can rescue the effects of capillary rarefaction. Using microsphere injections to induce random arteriolar blockade, we quantified the influence of a reduced number of perfused capillaries on muscle performance and hindlimb blood flow. Our findings suggest that capillary rarefaction is a major physiological contributor to skeletal muscle fatigability, and hence limits exercise tolerance, but that mechanical stimuli can successfully reverse pathological changes and restore muscle function to normal levels.

## Methods

### Ethical approval

All experimental work complied with the UK Animals (Scientific Procedures) Act 1986, and local approval was granted by the University of Leeds Animal Welfare and Ethical Review Committee (70/08674). All experiments conformed to the principals and regulations described by guidelines published in the *Journal of Physiology* (Grundy, 2015).

### Animal surgery: acute effects of microsphere injection

Anaesthesia in male Wistar rats [body mass (*M*_b_): 258 ± 16 g, range: 224–296 g, *N* = 25) was induced with isoflurane (4% in 100% O_2_) and subsequently maintained by constant alfaxalone (Alfaxan: Jurox, Crawley, UK) infusion (30–35 mg kg^−1^ h^−1^) delivered via an external jugular vein catheter. A tracheotomy tube allowed spontaneous breathing. A catheter (PE20) was implanted into the carotid artery to record heart rate and measure blood pressure with a pressure transducer (AD Instruments, Oxford, UK).

Spatially random occlusion of microvessels in the extensor digitorum longus (EDL) muscle was achieved through injection of polystyrene microspheres (Triton Technology Inc., San Diego, CA, USA) via a catheter (PE10 heat‐pulled to reduce tip size) implanted in a branch of the femoral artery (superficial epigastric artery). Microspheres (mean diameter 10 µm) stochastically blocked terminal arterioles, preventing blood flow in narrower downstream capillaries (Eriksson & Myrhage, 1972; Egginton & Hudlická, 1999) and thereby inducing acute functional microvascular rarefaction. No evidence was found in any sample of trapped microspheres in the capillary bed during histological analysis of capillary perfusion. Initial experiments indicated that injecting 10:1 dilutions of the microsphere suspension (as per the manufacturer's instructions and accepted practice in the field; Deveci & Egginton, 1999) induced a significant and persistent increase in local femoral blood flow. To reduce this bias, microspheres were centrifuged (number of microspheres  = 100,000–1,000,000; this range was found to be appropriate for inducing progressively more severe arteriolar occlusion), the supernatant was removed and washed spheres were re‐suspended in saline. This eliminated the confounding effect of the carrying solution on femoral artery vascular conductance (FVC) during the 10 min following injection.

### Animal surgery: chronic effects of arteriolar blockade

**Figure 1:**
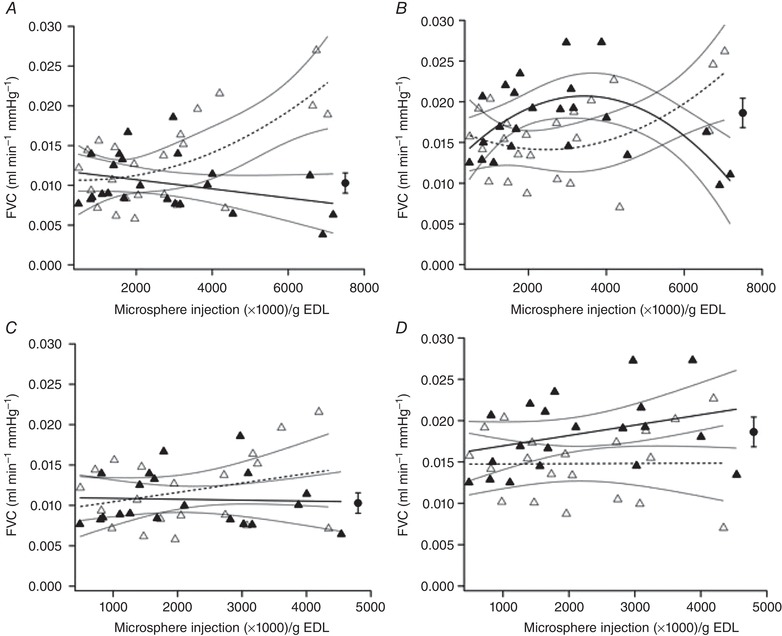
Effect of increasing acute microsphere dose (and consequently arteriolar occlusion) on femoral artery vascular conductance (FVC) during rest (*A*, *C*) and after 3 min of 10Hz stimulation (*B*, *D*) Ipsilateral (open triangles) and contralateral (filled triangles) data are shown in each panel. 95% confidence intervals are displayed around regression lines. There was a dose‐dependent effect on ipsilateral resting (*A*) (*r* ^2^ = 0.444, *P* = 0.003) and end‐stimulation (*B*) (*r* ^2^ = 0.304, *P* = 0.027) FVC. In the resting contralateral limb (*A*) there was a non‐significant relationship (*r* ^2^ = 0.103, *P* = 0.135) but decreasing end‐stimulation flow (*r* ^2^ = 0.393, *P* = 0.007) was detected with higher dose (*B*). Given the outliers observed at the highest doses (*A*, *B*), these data were omitted and the analyses re‐run. Omitting these confounding data (*C*, *D*) we found that there was no effect on resting (*C*) (ipsilateral: *r* ^2^ = 0.089, *P* = 0.200; contralateral: *r* ^2^ = 0.002, *P* = 0.864) or end‐stimulation (*D*) (ipsilateral: *r* ^2^ = 0.000, *P* = 0.974; contralateral: *r* ^2^ = 0.110, *P* = 0.153) FVC for mild to moderate arteriolar occlusion (*P* > 0.05). The effect of femoral ligation on contralateral FVC is also displayed (filled circle); no equivalent ipsilateral value was calculated due to cessation of hindlimb blood flow.

Muscle overload has been shown to precipitate an angiogenic response via upregulation of vascular endothelial growth factor (VEGF) and its main signalling receptor Flk‐1 in what is considered to be a mechanotransduction‐mediated response (Egginton *et al*. 2011, 1998; Williams *et al*. 2006). Therefore, unilateral overload to induce adaptive remodelling of the EDL was induced by surgical extirpation of the synergist, tibialis anterior (TA) (Zhou *et al*. 1998; Deveci & Egginton, 2002), in two new groups of animals. One group (OV: *M*_b_ at terminal experiment: 281 ± 20 g, range: 248–310 g, *N* = 8) underwent TA removal only, while in addition to TA removal, the other group (OV+MS: terminal *M*b: 283 ± 17 g, range: 261–305 g, *N* = 8) also received an injection of a moderate dose of microspheres (350,000; ascertained above) after removal of the TA, following the protocol used in acute experiments. A further group (chronic MS: terminal *M*_b_: 302 ± 9 g range: 290–315 g, *N* = 7) underwent only an injection of microspheres, which was of greater magnitude (700,000) to accommodate the probable reduced microsphere delivery to the EDL via blood flow to the TA. When transformed to a mass‐specific dose, this remained within the range at which resting blood flow remained unperturbed (∼4,762,000 microspheres per gram EDL, Fig. 1). Surgical procedures were undertaken in animals with body mass approximating that used in acute experiments. Data from intact control animals (terminal *M*_b_: 257 ± 15 g, range: 224–288 g, *N* = 15) was used for comparison. Surgical anaesthesia was induced and maintained by isoflurane inhalation. Post‐operative analgesia [buprenorphine (Vetergesic, Ceva, Amersham, UK) 0.05 mg kg^−1^] and antibiotic [enrofloxacin (Baytril, Bayer, Reading, UK) 2.5 mg kg^−1^] were given in all cases. Following a 14‐day recovery period, EDL fatigue resistance and hindlimb blood flow were quantified.

### Quantifying muscle performance and hindlimb perfusion

Bilateral EDL twitch force was measured simultaneously by connecting the muscle of each limb to a lever arm force transducer system (305B‐LR: Aurora Scientific, Aurora, ON, Canada), following extirpation of the synergist TA to allow unimpeded access to the EDL. The EDL was indirectly stimulated with electrodes adjacent to the popliteal nerve (Hudlická *et al*. 1977). To measure femoral artery blood flow throughout the experiment, perivascular flow probes (0.7PSB; Transonic, Ithaca, NY, USA) were placed at the proximal aspect of the profunda femoris artery bifurcation. Before fatigue measurements, muscle length and current delivery were set to ensure maximal isometric twitch force production. Resistance to fatigue was quantified in the EDL whereby a 30 s period of 1 Hz twitches to activate the metabolic machinery was followed by a fatigue test (Egginton & Hudlická, 1999) during which the muscle was stimulated for 180 s with 10 Hz twitches (0.3 ms pulse width, supramaximal voltage).

Following this initial bout of stimulation to establish reference fatigue curves and blood flow responses, a saline solution (0.6 ml) containing microspheres was injected to randomly occlude arterioles in the EDL. To ensure that the bulk of flow from the femoral artery passed through the EDL, minimising blockage of arterioles in other limb muscles from systemic application (verified by histology in pilot studies, data not shown), functional hyperaemia was induced in the EDL before and after injection by 30 s of 4 Hz stimulation (0.3 ms pulse width, supramaximal voltage). The number of injected microspheres was within the range 100,000–1,400,000, and is expressed as microspheres per gram of EDL. After a post‐injection period of at least 10 min, during which time all variables (blood pressure, heart rate, femoral blood flow) returned to stable baseline levels, the protocol for inducing fatigue was repeated to establish the acute effects of reduced microvascular perfusion on muscle performance. Pilot studies demonstrated that a second stimulation did not significantly affect blood flow or fatigue resistance in muscles with an intact microcirculation. To assess the effects of major microvascular blockade in another group of animals the proximal femoral artery was ligated. Muscle force, blood flow and pressure data were acquired using PowerLab 8/35 and analysed in LabChart 8 (both from AD Instruments).

A fatigue index (FI) was calculated as muscle tension at the end of stimulation/peak muscle tension at the start of the test, using the average of five consecutive twitches. Relative FI was calculated for each muscle as the ratio of pre‐ to post‐microsphere injection or ligation values. FVC was calculated as femoral blood flow/mean arterial pressure. Reported FVC was normalised for EDL mass to account for muscle hypertrophy in chronically treated animals. Hyperaemic scope was calculated as end‐stimulation FVC/resting FVC.

Following the final fatigue stimulation, capillary plasma perfusion was visualised by an arterial injection of a fluorescein isothiocyanate (FITC)‐labelled dextran solution (50 mg kg^−1^, MW = 500,000; Sigma, Poole, UK) while the muscle was stimulated supramaximally at 4 Hz to ensure the FITC dextran had full vascular access to the EDL. Whether these ‘functional’ (i.e. perfused) capillaries were able to support red blood cell flux was not explicitly quantified. Muscles were left *in situ* for at least 1 min after stimulation to ensure appearance of dye in capillaries (Snyder *et al*. 1992) and animals were then killed by a Schedule 1 method.

### Histology

Immediately following death of the animal, the EDL muscle was excised, blotted dry and weighed. The muscle was then mounted in OCT embedding medium (Thermo Scientific, Loughborough, UK), frozen in liquid nitrogen‐cooled isopentane and stored at −80°C. In a subset of animals, in 10 µm cryostat sections capillary boundaries were labelled with a carbohydrate‐binding protein known to identify rodent endothelial cells (*Griffonia simplicifolia* lectin I, 5 µl ml^−1^ FL‐1101 GSL I; Vector Labs, Peterborough, UK). To identify muscle fibre types, serial sections were blocked with 10% goat serum (Vector Labs) in PBS for 60 min, then incubated for 120 min with monoclonal antibodies BAD‐5 (1:600), SC‐71 (1:600), 6H1 (1:50) and BF‐F3 (1:100) in blocking solution for types I, IIa, IIx and IIb, respectively (Developmental Studies Hybridoma Bank, Iowa City, IA, USA). After washing in PBS three times for 5 min, sections were incubated in secondary antibodies Alexa Fluor 350 IgG2b for type I (1:500), Alexa Fluor 488 IgG1 for type IIa (1:500) and Alexa Fluor 555 IgM for types IIb and IIx (1:500) (Thermo Fisher Scientific Inc., Waltham, MA, USA) in blocking solution in the dark. After being washed once more, the slides were mounted using ProLong Diamond Antifade mountant (Thermo Fisher Scientific). Digital images of whole‐muscle cross‐sections were acquired at ×20 magnification using a confocal microscope (Leica TCS SP5). Subsequent image analysis with BTablet and AnaTis (BaLoH Software, Ooij, The Netherlands) enabled calculation of a capillary perfusion index (perfused/unperfused vessels). Three regions of interest (475 × 475 µm^2^) on the muscle image were used to establish an unbiased counting frame with which to quantify muscle fibre type composition, capillarisation and capillary supply (domain) area, taking into account the regional heterogeneity of capillary distribution and regional differences in fibre type composition (Kissane *et al*. 2018). Regions of interest were spaced equidistantly across the medial‐lateral axis of the muscle in a systematic random manner, namely without *a priori* determination of placement. Capillary localisation depended upon staining intensity and anatomical landmarks; to be identified as a capillary, staining had to be at a location where one can expect a capillary and be elevated above the surrounding stain intensity. Image processing using the thresholding function in ImageJ (Schneider *et al*. 2012) allowed removal of non‐specific staining that was below the high‐intensity staining of identified capillaries. Pilot experiments indicated that FITC migration from capillaries was a minor problem. A small degree of FITC extravasation occurred in stored samples but observations show this to be greater if sections rather than tissue blocks are stored. We therefore adopted a protocol whereby block storage was the preferred approach until batch analysis was possible, at which time sections were cut and quickly imaged. Estimation of the oxygen partial pressure distribution within representative EDL histological sections was performed in a custom MATLAB program (‘oxygen transport modeler’) (Al‐Shammari *et al*. 2019). Model assumptions include estimates of capillary radius (1.8–2.5 × 10^−4^ cm), muscle oxygen demand (15.7 × 10^−5^ ml O_2_ ml^−1^ s^−1^), myoglobin concentration (10.2 × 10^−3^ ml O_2_ ml^−1^), O_2_ solubility (3.89 × 10^−5^ ml O_2_ ml^−1^ mmHg^−1^) and diffusivity (1.73 × 10^−7^ cm^2^ s^−1^) (Al‐Shammari *et al*. 2019). No direct measurement of these parameters was possible in this study, so these values were applied across all groups. Although we have adopted a modelling approach to facilitate group comparison, physiological data to suggest *P*O_2_ buffering by myoglobin in skeletal muscle (Gayeski *et al*. 1985; Gayeski & Honig, 1986) supports an alternative perspective on muscle–capillary oxygen diffusivity, whereby tissue oxygenation is not directly influenced by diffusion distance (Hepple *et al*. 2000), contrary to classical theory (Krogh, 1919). Whilst the considerable technical challenge of accurately measuring dynamic quantities (including *P*O_2_) may confound the interpretation of these empirical data (Groebe, 1996), it is clear that this remains a controversial topic; nevertheless, while recognising its inherent methodological limitations, we consider it timely to apply a model that assumes *P*O_2_ gradients to be a cardinal factor in determining muscle oxygenation, as it allows for theoretical exploration of oxygen flux during simulated perturbations in metabolic demand and altered capillary supply.

### Statistical analyses

To test for an effect on FVC of injections of saline, microsphere dose and microsphere solution, an ANOVA with Tukey *post hoc* tests was performed. Associations between microsphere dose, muscle performance, FVC and histological parameters were assessed using ordinary least‐squares regression. Mean arterial pressure before and after microsphere administration was assessed using paired‐samples *t* tests. ANOVA with Tukey *post hoc* tests were used to determine whether chronic differences occurred after surgical intervention. Data are presented as mean ± SD. Statistical analyses were performed in SPSS (v. 25) and differences and correlations were considered significant at *P *< 0.05.

## Results

### Immediate effect of microsphere injections on FVC

No difference in resting FVC was observed before injection of saline, washed microspheres or microsphere solution (*P* = 0.544). FVC was increased in animals injected with microspheres in the normal solution after 2 min (*P* = 0.001) and 5 min (*P* = 0.006), returning to parity after 10 min (*P* = 0.208), compared to animals injected with saline or washed microspheres. There was no difference between FVC following injections of saline and washed microspheres (2 min: *P* = 0.371; 5 min: *P* = 0.952). Contralateral FVC was unaffected throughout (rest: *P* = 0.752; 2 min: *P* = 0.987; 5 min: *P* = 0.965; 10 min: *P* = 0.208).

### Acute effects of arteriolar blockade on FVC

There were no significant bilateral differences in mean femoral blood flow at rest (ipsilateral 1.19 ± 0.26, contralateral 1.22 ± 0.27 ml min^−1^, *P* = 0.784) or at the end of stimulation (ipsilateral 1.71 ± 0.39, contralateral 1.86 ± 0.35 ml min^−1^, *P* = 0.380) before injection of microspheres. Peak systolic femoral flow during rest (*P* = 0.019) and flow amplitude (the difference between maximum and minimum flow recorded at systole and diastole) increased with microsphere dose (*P* = 0.045); in contrast, at end‐stimulation there was no effect of dose (*P* > 0.05). There was no significant effect of dose or microsphere administration itself on resting (control: 123 ± 10, after injection: 122 ± 12 mmHg, *P* = 0.282) or end‐stimulation mean arterial pressure (control: 119 ± 15, after injection: 118 ± 15 mmHg, *P* = 0.478). Resting (control: 12.2 ± 8.2, after injection: 13.8 ± 8.4 mmHg, *P* = 0.664) and end‐stimulation (control: 13.0 ± 8.4; after injection: 13.9 ± 8.8 mmHg, *P* = 0.742) pulse pressures were similarly unaffected by dosage.

At higher doses than 100,00–700,000 microspheres there was a positive relationship between dose and FVC that was best described by a polynomial function both at rest (Fig. 1*A*) and end‐stimulation (Fig. 1*B*). Contralateral FVC (Fig. 1*A*) was unaffected at rest, but the ipsilateral trend was paralleled at end‐stimulation (Fig. 1*B*). Interestingly, the highest dose of microspheres elicited a persistent increase in resting FVC; omitting these data (*N* = 3) indicated that FVC was unaffected at lower doses (Fig. 1*C*, *D*). Therefore, it appears that resting and active FVC is enhanced by the magnitude of microsphere dose above a threshold value.

### Chronic effects of arteriolar blockade on FVC

**Figure 2:**
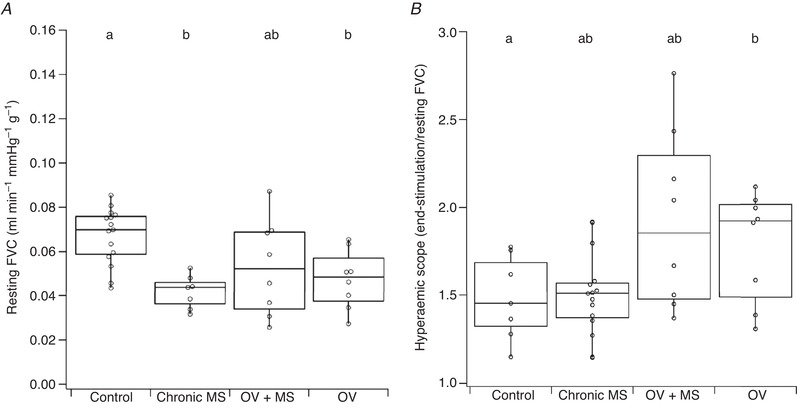
Femoral artery vascular conductance (FVC) during rest and hyperaemic scope after 3 min of 10Hz stimulation Reduced resting FVC (normalised to muscle mass) was observed after chronic arteriolar blockade (chronic MS) and overload (OV). The magnitude of functional hyperaemia was equivalent to the control group in chronic MS and overload with microspheres (OV+MS), but enhanced after overload (OV). Unmatched lower‐case letters denote statistical significance (*P* < 0.05).

Muscle overload (OV) induced a reduced resting FVC normalised to muscle mass (Fig. 2*A*; *P* = 0.015). Two weeks after microsphere (MS) injection, resting FVC was lower than control (*P* = 0.011), but in OV+MS no decrease was observed (Fig. 2*A*; *P* = 0.147). The fractional increase in FVC (i.e. magnitude of functional hyperaemia) during stimulation was enhanced in OV (*P* = 0.029; Fig. 2*B*) while chronic application of microspheres did not have a significant effect on the hyperaemic response (chronic MS: *P* = 0.999; OV+MS: *P* = 0.217; Fig. 2*B*, Table 1).

**Table 1:** EDL fatigue resistance and blood flow (normalised to muscle mass) during rest and stimulation

		Control	Chronic MS	OV+MS	OV
Fatigue index		0.47 ± 0.09^a^	0.36 ± 0.03^b^	0.65 ± 0.11^c^	0.64 ± 0.08^c^
Maximum twitch tension (N)		0.35 ± 0.10^a^	0.35 ± 0.07^a^	0.52 ± 0.10^b^	0.48 ± 0.08^b^
Maximum twitch tension per g EDL (N g^−1^)		2.41 ± 0.64^ab^	1.90 ± 0.44^a^	2.72 ± 0.60^b^	2.54 ± 0.40^ab^
Mean femoral flow (ml min^−1^ g^−1^)	Rest	8.13 ± 1.62^a^	5.37 ± 1.12^b^	6.38 ± 2.77^ab^	5.58 ± 1.47^b^
	End‐stimulation	11.70 ± 2.49^a^	7.81 ± 1.90^b^	10.56 ± 3.46^a^	10.44 ± 1.09^a^
Peak (systolic) femoral flow (ml min^−1^ g^−1^)	Rest	27.00 ± 6.79^a^	14.82 ± 2.63^b^	20.52 ± 5.24^b^	17.64 ± 4.17^b^
	End‐stimulation	34.46 ± 8.24^a^	19.26 ± 5.39^b^	29.48 ± 7.83^a^	28.20 ± 6.51^ab^
Flow amplitude (ml min^−1^ g^−1^)	Rest	25.22 ± 7.00^a^	13.39 ± 2.92^b^	18.95 ± 4.80^ab^	16.50 ± 4.25^b^
	End‐stimulation	31.40 ± 8.45^a^	16.46 ± 5.26^b^	25.60 ± 8.05^ab^	24.92 ± 7.13^ab^
FVC (ml min‐^1^ mmHg^−1^ g^−1^)	Rest	0.067 ± 0.013^a^	0.042 ± 0.008^b^	0.053 ± 0.022^ab^	0.047 ± 0.013^b^
	End‐stimulation	0.100 ± 0.020^a^	0.062 ± 0.016^b^	0.089 ± 0.024^a^	0.086 ± 0.015^ab^
	Hyperaemic scope	1.50 ± 0.24^a^	1.48 ± 0.24^ab^	1.79 ± 0.31^ab^	1.92 ± 0.51^b^

Unshared superscript letters denote statistical significance (*P* < 0.05) as assessed by Tukey *post hoc* tests.

### Muscle fatigue

**Figure 3:**
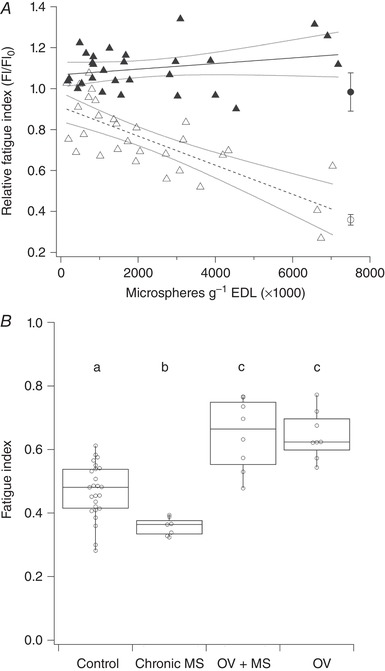
Change in muscle fatigue index (FI) with acute (*A*) and chronic (*B*) microvascular occlusion and overload *A*, there was a significant decrease in ipsilateral relative FI (i.e. pre‐injection/post‐injection FI) with increasing dose of microspheres injected (open triangles, dashed line: *r* ^2^ = 0.570, *P* < 0.001) while there was no effect on the contralateral FI (filled triangles, continuous line) (*r* ^2^ = 0.067, *P* = 0.182). The effect of femoral artery ligation is displayed for ipsilateral (open circle) and contralateral (filled circle) extensor digitorum longus muscle. *B*, chronic occlusion of arterioles by microspheres (chronic MS) caused impaired FI (*P* = 0.016) relative to control while both overload with microspheres (OV+MS) and overload (OV) improved FI (*P* < 0.001). Unmatched lower‐case letters denote statistical significance (*P* < 0.05).

In the acute model, before injection there was no bilateral difference in the fatigue index (FI) of EDL (ipsilateral: 46.9 ± 8.5%, *N* = 25; contralateral: 48.5 ± 8.4%; *N* = 24, *F* = 0.477, *P* = 0.493). A significant, dose‐dependent, inverse relationship between the number of microspheres injected and relative FI was detected in the ipsilateral (*P* < 0.001), but not in the contralateral control EDL (*P* = 0.182). The FI in rats with ligated femoral artery was significantly impaired compared to simultaneously measured contralateral FI (*P* < 0.001) (Fig. 3*A*).

FI was improved in OV (*P* < 0.001) but was impaired compared to control values in chronic MS (*P* = 0.016; Fig. 3*B*, Table 1). In contrast, OV+MS had enhanced FI (*P* < 0.001). There was no difference between OV and OV+MS FI (*P* > 0.05), but in chronic MS animals FI was significantly impaired compared to OV+MS (*P* < 0.001; Table 1, Fig. 3*B*).

### Histology

**Figure 4:**
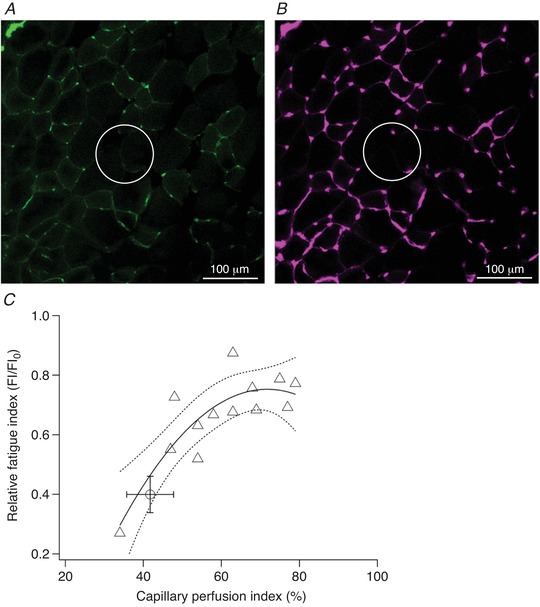
Histological quantification of microsphere‐injected extensor digitorum longus (EDL) Dextran‐FITC‐labelled perfused (*A*) and total (*B*) capillary supply after moderate microsphere injection. Comparison of circled regions highlights functional microvascular rarefaction as capillaries remain unperfused after arteriolar blockade. There was a significant polynomial relationship (*r* ^2^ = 0.720, *P* = 0.002) between ipsilateral capillary perfusion index (i.e. proportion of total capillaries that were perfused) and relative fatigue index (FI) in animals undergoing acute injection of microspheres (*C*). The effect of femoral ligation is also shown for comparison (open circle). Regression line and 95% confidence intervals are plotted.

**Figure 5:**
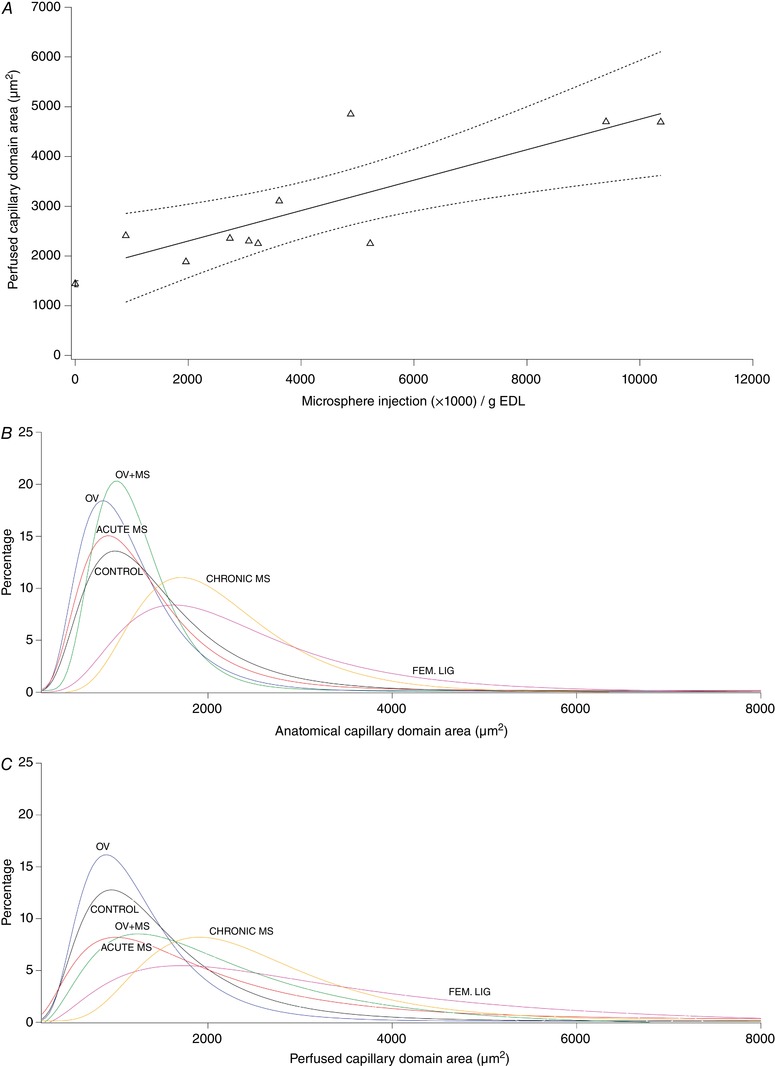
Effects of acute (*A*) and chronic (*B*, *C*) microsphere injection and overload on total (*B*) and perfused (*A*, *C*) capillary domain area *A*, acute perturbation in capillary domain area (CDA) associated with microsphere injection. Mean domain area increases with arteriolar occlusion. Domain size increased (*r* ^2^ = 0.632, *P* = 0.006) with increasing microsphere dosage and consequent arteriolar occlusion. Mean ± SEM control value is also shown (far left). *B* and *C*, lognormal frequency distribution of capillary domain area (exemplar data from each group). Capillary domain areas were calculated for each capillary in a sample and each was binned into one of an incrementally increasing group, each with a range of 200 µm^2^ (e.g. 201–400 µm^2^, 401–600 µm^2^ etc). The representation of each domain area size in the muscle was calculated as a percentage of total domains analysed. The effects of acute (medium dose of microspheres, equivalent to the chronic injections) and chronic distributed ischaemia and overload are displayed. Overload precipitates a narrowing of the total CDA curve indicating that domain sizes occur across a smaller range, which is consistent with an angiogenic response. Acute and chronic application of microspheres causes a downwards shift in the curve, indicating that there is a greater distribution of domain sizes as larger capillary domain areas are precipitated by arteriolar blockade. There was a rightwards shift in the curve for total and perfused CDA in chronic MS and femoral ligation animals, corresponding to an undesirable increased mean domain area and consequently a longer average diffusion distance to and from the capillary.

A significant positive relationship was found between capillary perfusion and FI (*P* = 0.002), indicating that a reduction in perfusion after microsphere injection was associated with declining muscle performance (Fig. 4). No association existed between contralateral capillary perfusion and FI (*r* ^2^ = 0.038, *P* = 0.591), indicating that potential microsphere spill‐over to the contralateral limb was negligible. Acute microsphere injection resulted in significant dose‐dependent increases in perfused capillary domain area (CDA) (*P* = 0.006; Figure 5a) and heterogeneity of capillary spacing (logSD) (*P* = 0.025). Compared to controls, overload caused ipsilateral muscle hypertrophy (ipsilateral/contralateral EDL mass) of 19% (*P* < 0.001) while no change in muscle mass occurred in chronic MS (*P* = 0.705; Table 2). Total capillary density (CD) was higher after overload than for control and chronic MS (*P* < 0.01; Table 2). There was no difference in total (anatomical) CD between OV and OV+MS (*P* = 0.969). An increased anatomical CDA was measured following chronic microsphere injections *(P* < 0.01); in contrast, relatively large data variability resulted in no statistical difference for perfused CDA between groups (Table 2, Fig. 5). The heterogeneity of capillary spacing was unaffected by overload and chronic microsphere injection (*P* > 0.05). No differences were found between groups in EDL fibre‐type composition (Type I: *P* = 0.935; Type IIa: *P* = 0.821; Type IIb/IIx: *P* = 0.950) (Table 2). In contrast, overload caused an increased cross‐sectional area of Type IIa and IIb/IIx fibres (Table 2).

**Table 2:** Morphometric characteristics of EDL

	Control	Chronic MS	OV+MS	OV
EDL mass (mg per g *M*_b_)	0.57 ± 0.05^a^	0.59 ± 0.06^a^	0.68 ± 0.06^b^	0.67 ± 0.03^b^
Bilateral EDL mass hypertrophy	1.02 ± 0.09^a^	1.06 ± 0.010^a^	1.19 ± 0.07^b^	1.19 ± 0.06^b^
Anatomical capillary density (mm^2^)	617 ± 61^a^	556 ± 63^a^	813 ± 114^b^	836 ± 131^b^
Perfused capillary density (mm^2^)	564 ± 107^a^	420 ± 95^b^	512 ± 48^ab^	728 ± 165^c^
Anatomical capillary: muscle fibre	1.47 ± 0.30^ab^	1.29 ± 0.13^a^	1.66 ± 0.19^b^	1.63 ±0.22^b^
Perfused capillary: muscle fibre	1.25 ± 0.29^ab^	0.96 ± 0.19^a^	0.92 ± 0.18^a^	1.42 ± 0.31^b^
Anatomical CDA (µm^2^)	1399 ± 156^a^	1841 ± 204^b^	1414 ± 158^a^	1337 ± 155^a^
Perfused CDA (µm^2^)	1854 ± 397^a^	2506 ± 765^a^	2274 ± 519^a^	1759 ± 521^a^
Anatomical log_rSD_	0.107 ± 0.008^a^	0.096 ± 0.010^a^	0.100 ± 0.012^a^	0.104 ± 0.008^a^
Perfused log_rSD_	0.127 ±0.016^a^	0.109 ± 0.008^a^	0.112 ± 0.019^a^	0.112 ± 0.014^a^
Type I (%)	3.8 ± 2.1^a^	3.9 ± 2.2^a^	3.3 ± 1.8^a^	3.5 ± 2.4^a^
Type 1 FCSA (µm^2^)	922 ± 217^a^	1008 ± 138^a^	1042 ± 180^a^	1010 ± 173^a^
Type IIa (%)	21.4 ± 5.0^a^	19.9 ± 5.4^a^	21.5 ± 4.2^a^	23.2 ± 6.2^a^
Type IIa FCSA (µm^2^)	933 ± 99^a^	1012 ± 133^ab^	1169 ± 120^b^	1113 ± 120^b^
Type IIb/IIx (%)	74.8 ± 6.9^a^	76.2 ± 7.3^a^	75.1 ± 4.2^a^	73.3 ± 8.5^a^
Type IIb/IIx FCSA (µm^2^)	1644 ± 189^a^	2085 ± 310^b^	2269 ± 182^b^	2121 ± 223^b^

Abbreviations: CDA, capillary domain area; FCSA, fibre cross‐sectional area. Superscript letters denote statistical significance (*P *≤ 0.05) as assessed by Tukey *post hoc* tests.

## Discussion

The main observations of the present study are that (1) the extent of capillary rarefaction is correlated with muscle fatigue resistance, independent of changes to arterial blood flow, (2) capillary rarefaction did not attenuate the hypertrophic response to an overload stimulus, and (3) overload‐induced angiogenesis alleviated the decline in muscle performance.

### Effect of capillary rarefaction on muscle performance

Our novel approach provides insight into the effects of up to 70% capillary occlusion on muscle performance. This range encompasses the 32% reduction in the number of capillaries per muscle fibre reported in muscles of CHF patients (Duscha *et al*. 1999). The strong relationship between muscle fatigue resistance and functional (perfused) capillaries (Fig. 4*C*) is indicative of the critical function of the microcirculation, as also observed in cardiac muscle (Hauton *et al*. 2015). Our findings may be particularly relevant to the development of heart failure, where the magnitude of capillary rarefaction in skeletal muscle progressively increases with time after experimental induction of the condition (Nusz *et al*. 2003), and where the decline in fatigue resistance depends upon clinical severity (Buller *et al*. 1991). Considered together with the angiogenic‐driven improvement in muscle fatigue resistance that follows long‐term stimulation (Hudlická *et al*. 1977) and mechanical overload (Tables 1 and 2, Fig. 3*B*) (Deveci & Egginton, 2002), there is strong evidence to indicate that maintaining adequate capillarisation is a component that determines optimal muscle function, and that rarefaction is a significant contributory factor to reductions in exercise tolerance in disease (Duscha *et al*. 1999; Nusz *et al*. 2003).

Our current understanding of the effects of peripheral disturbances in blood flow distribution on skeletal muscle performance, independent of confounding variables seen with co‐morbidities, is largely based on the iliac/femoral artery ligation model (Lotfi *et al*. 2013), which induces a comprehensive restriction on hindlimb perfusion (Couffinhal *et al*. 1998; Hudlická *et al*. 2008). In this model, fatigue resistance of skeletal muscle is initially compromised due to the constraint on arterial flow and corresponding limitation on the supply of substrates to, and removal of heat and waste products from, active muscle tissue, with subsequent improvements coincident with development of collateral circulation (Couffinhal *et al*. 1998; Hudlická *et al*. 2008; Hellingman *et al*. 2010). However, this approach does not consider the more subtle effects of capillary rarefaction, which result in localised pockets of ischaemia (as indicated by increased capillary supply area while capillary distribution is unaffected), within muscle tissue, on skeletal muscle performance independent of blood flow, as may occur in heart failure patients (Nusz *et al*. 2003). Random blockage of arterioles in the current study (Fig. 4) demonstrates that at all but the lowest microsphere doses, acute reductions in functional capillary supply, which would be impossible to imitate using established ligation models (Lotfi *et al*. 2013), have a negative impact on skeletal muscle performance (Fig. 4*A*). We can thus simulate the early stages of microvascular disease in skeletal muscle, where limited changes in the microcirculation occur without affecting arterial perfusion, which may also become impaired with disease progression (Lejemtel *et al*. 1986). It is important to be cautious in extrapolating from our findings in the context of therapeutic intervention because the animals used in these experiments were young adults; diseases that affect the microcirculation including CHF and diabetes mellitus typically occur in more elderly humans. However, the basic relationships established would probably be enhanced by such co‐morbidities. For example, in addition to microvascular rarefaction (Nusz *et al*. 2003), impaired capillary red blood cell flow (Kindig *et al*. 1999; Richardson *et al*. 2003), muscle wasting (Fulster *et al*. 2013), impaired functional hyperaemia (Sullivan *et al*. 1989) and reduced oxidative capacity (Bowen *et al*. 2015; Southern *et al*. 2015) all contribute to suboptimal muscle performance in disease. The data presented in this paper present the effects of microvascular rarefaction without such concomitant changes in healthy tissue and therefore provide insight into the role of the microcirculation for muscle function.

**Figure 6:**
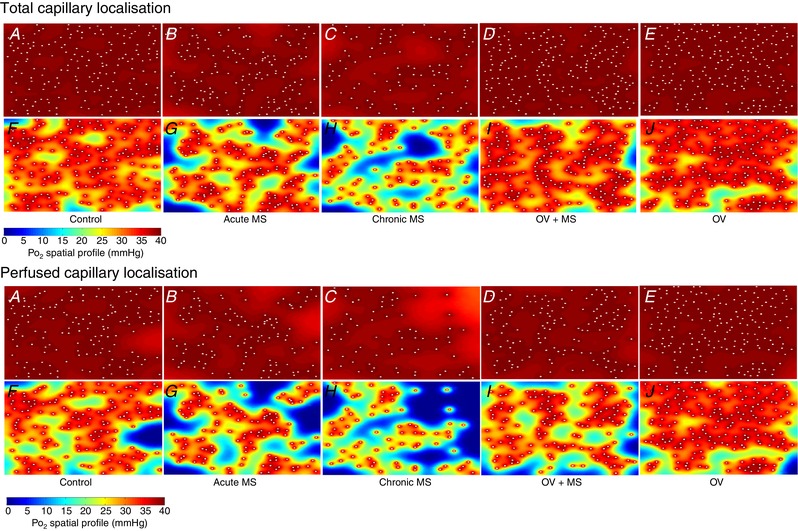
Oxygen transport modelling Simulation of muscle *P*O_2_ at rest (*A–E*) and maximal rate of oxygen consumption (*F–J*) in representative images. Acute effects (*B* and *G*) are seen after injection of 200,000 microspheres. Model assumptions are provided in Methods. Areas of muscle hypoxia (highlighted in blue, where *P*O_2_ < 0.5 mmHg) during exertion are increased after microvascular blockade (*G* and *H*) and this profile corresponds to an attenuated fatigue resistance.

Interestingly, the relationship between FI and functional capillary density (Fig. 4) indicates that skeletal muscle is sensitive to small perturbations in the microcirculation, although to a lesser extent than cardiac muscle (Hauton *et al*. 2015), which has a correspondingly highly oxidative metabolic phenotype (De Sousa *et al*. 2002). This suggests that there is little functional reserve in the capillary bed, i.e. a small degree of rarefaction (*c*. 10%; Fig. 4*C*) can be observed without deleterious effects on muscle performance but there is a steep decline thereafter. Indeed, theoretical (Al‐Shammari *et al*. 2014) models of oxygen transport within muscle and experimental studies of muscle structure (Degens *et al*. 2006) have demonstrated that an optimal distribution of capillaries exists, and any disturbance to this pattern only decreases intramuscular *P*O_2_ (Fig. 6). Increased capillary domain areas with distributed ischaemia may therefore impose a restriction on muscle performance as oxygen delivery becomes spatially limited (Figs 5 and 6). Consequently, compensatory mechanisms to ameliorate local ischaemia, such as microvascular dilatation to locally maximise flow (Klitzman *et al*. 1982), appear inadequate in maintaining muscle performance in otherwise healthy tissue.

The EDL contains oxidative and glycolytic fibres (Table 2) (Pullen, 1977; Kissane *et al*. 2018) and localised ischaemia may particularly constrain oxidative metabolism in Type I and IIa fibres, contributing to increased fatigability. Random blockade of arterioles is predicted to disrupt the spatial distribution (Fig. 5, Table 2) of perfused capillaries, as was indeed observed in the acute model, potentially contributing to declining muscle performance due to reduced tissue oxygenation (Greene *et al*. 1989; Degens *et al*. 2006; Goldman *et al*. 2006; Al‐Shammari *et al*. 2014). However, no statistical difference in heterogeneity of capillary spacing was found between chronic models, indicating that the random arteriolar blockade resulted in a stochastic loss of functional capillaries. Increased total CDA after chronic microsphere injection is indicative of a greater diffusion distance across respiring muscle tissue and while the equivalent perfused CDA (Table 2) is not statistically different from other groups, the trend to an increased mean value suggests a developing biological significance with a limitation on oxygen delivery that is likely to contribute to reduced fatigue resistance (Al‐Shammari *et al*. 2014). Interestingly, no shift in fibre‐type composition was detected after chronic arteriolar blockade or overload (Table 2), indicating that the observed reductions in fatigue resistance after microsphere injection were principally driven by microvascular disruption. In CHF, other factors such as a slow‐to‐fast shift in fibre type composition (Drexler *et al*. 1992) may compound the effects of capillary rarefaction, and together with fibre atrophy aggravate the capillary rarefaction‐induced reductions in muscle fatigue resistance. Effective therapeutic interventions may thus lie in the development of exercise training protocols that prevent or reverse capillary rarefaction, as seen in animal models of hypertension (Amaral *et al*. 2001, 2000; Melo *et al*. 2003; Fernandes *et al*. 2012) and in heart failure patients (Gustafsson *et al*. 2001; Eleuteri *et al*. 2013).

Based on our acute model (Fig. 3*A*), the moderate volume of microspheres injected in chronic experiments was expected to impose a deterioration in FI of ∼30%. This impairment persists even 2 weeks after injection in chronic MS, indicating that little or no angiogenesis occurred to rectify the loss of functional capillaries and consequent loss of function (Tables 1 and 2, Fig. 3B). This contrasts with the upregulation of capillary growth during chronic systemic hypoxia (Deveci *et al*. 2001, 2002), suggesting that compensatory mechanisms to overcome microvascular constraint, even in otherwise healthy animals, are insufficient to recover function in the longer term. Note that contrasting results for exposure to chronic hypoxia have been reported (Sillau & Banchero, 1977; Snyder *et al*. 1985; Bigard *et al*. 1991), although methodological differences preclude direct comparison (see Deveci *et al*. 2001). However, persistently reduced FI in chronic MS is surprising as partial recovery occurs after extensive reduction in femoral flow following ligation (Hudlická *et al*. 2008). Arterial occlusion may therefore be a more potent stimulus for capillary proliferation than finer‐scale distributed ischaemia, suggesting wide‐scale tissue hypoxia stimulates a greater angiogenic response than local pockets of tissue hypoxia. Importantly, hypertrophic angiogenic remodelling of muscle was not diminished and was accompanied, as seen in other studies (Degens *et al*. 1993; Egginton *et al*. 2011, 1998; Zhou *et al*. 1998; Deveci & Egginton, 2002; Ballak *et al*. 2016), with enhanced resistance to fatigue after overload (Fig. 3*B*, Table 1), regardless of microvascular impediment. This indicates that an underlying capacity for microvascular regeneration can be harnessed given a mechanical stimulus of sufficient intensity. A similar increase in capillarity occurs with overload after arterial ligation (Deveci & Egginton, 2002), highlighting the potency of blood flow‐independent, mechanical stimuli for recovery of muscle capillarity and performance that could be harnessed in rehabilitation programmes.

### Hindlimb perfusion and capillary rarefaction

In the acute microsphere model, resting and end‐stimulation hindlimb blood flow were not adversely influenced by increasing arteriolar blockade (Fig. 2). In fact, resting blood flow was slightly elevated following administration of the highest dose, which may reflect a reactive hyperaemic response (Williams & Segal, 1993) to overcome local muscle ischaemia. Whatever the cause, the overall resting and contraction‐induced muscle blood flow was not limited by arteriolar blockade in the short term, and the decline in muscle fatigue resistance was thus probably related to diffusion limitations, as indicated by increased capillary domain size (Table 2, Fig. 5), rather than insufficient perfusion via the feed artery blood flow. The similarity in FI after both the highest dose of microspheres and femoral artery ligation is therefore informative because, despite comparatively high blood flow in the microsphere model, muscle performance is impaired due to reduced functional capillary density (Fig. 4*C*). In contrast, reduced arterial blood flow delivery rather than microvascular deficit determines the reduced performance of muscle in the ligation model. Interestingly, the femoral artery blood flow waveform was affected by rarefaction, with increased resting flow amplitude and peak flow associated with cardiac systole in more severe microvascular rarefaction. These observations in part agree with a theoretical model that predicted elevated blood flows but a smoother waveform with vessel rarefaction (Olufsen *et al*. 2012). We speculate that as arteriolar blockade increases, a femoral blood flow response is stimulated in an attempt to alleviate the increased ischaemic areas within the muscle.

In the chronically ischaemic hindlimb, skeletal muscle fatigability is considerably increased despite the number of capillaries per muscle fibre remaining undisturbed (Hudlická *et al*. 2008), highlighting the damaging influence of long‐term restricted blood flow. Thus, depressed exercise‐induced limb blood flow in disease may exacerbate the effect of capillary rarefaction on fatigue resistance by imposing a further convective constraint of oxygen delivery and waste product removal (Wilson *et al*. 1984). Dysfunction of the microcirculation by development of non‐ and slow‐flowing capillaries (Kindig *et al*. 1999; Richardson *et al*. 2003; Padilla *et al*. 2006) that is apparent in heart failure and diabetes is expected to compound this rarefaction‐derived constraint on performance by further reducing the functional capillary density.

The absence of a chronic effect on hyperaemia after microsphere administration (Table 1, Fig. 3*B*) indicates that no constraint was imposed upon the upregulation of femoral artery perfusion during stimulation, although a significantly lower end‐stimulation flow rate was measured (Table 1), which parallels the attenuated blood flow measured during gradually induced peripheral ischaemia (Tang *et al*. 2005). The amplitude of blood flow did not differ between chronic groups (Table 1), mirroring the similar mean arterial pressures and indicating the minimal effect of capillary rarefaction on upstream haemodynamics. No effect on resting blood flow (normalised to muscle mass) was reported in earlier overload experiments (Egginton *et al*. 1998) so it is unclear why this parameter was reduced in our chronic models (Table 1), although factors such as ambient temperature (He *et al*. 2002) may have exerted an influence. Nevertheless, this serves to highlight that improvement in FI after overload is independent of increased blood flow, and is instead driven by mechanical stimuli acting on muscle fibres and their vascular supply (Egginton *et al*. 1998; Egginton *et al*. 2011; Deveci & Egginton, 2002). Enhanced functional hyperaemia relative to control occurred in OV (Table 1), while peak flow did not differ from control values (Table 1), in agreement with previous data (Egginton *et al*. 1998). OV+MS had a similar hyperaemic response and peak flow values to OV, and peak flow was increased relative to chronic MS (Table 1). Note that the functional hyperaemia reported herein is relatively low when compared to that observed in the EDL (hyperaemic scope: 5.7) during unanaesthetised maximal exercise (Armstrong & Laughlin, 1985), indicating that there remained a potential for further increases in flow. Nevertheless, improvements in the capacity for upregulating arterial flow, as well as microvascular density, were elicited by overload and overcame any potential limitations on mean flow associated with chronic microvascular rarefaction.

Computational models predict that the magnitude of vessel rarefaction determines the scale of increased vascular resistance (Greene *et al*. 1989) and reduced muscle blood flow (Heuslein *et al*. 2015). Placement of femoral artery flow probes distal to the profunda femoris enabled gross hindlimb blood flow to be quantified, but the proportion of flow drawn by EDL was not explicitly measured. Furthermore, up to 30% of capillaries remained perfused after acute ligation, indicating that collateral artery blood flow prevented total (‘no‐flow’) muscle ischaemia (thus avoiding profound hypoxia). Quantification of individual muscle blood flow (Degens *et al*. 1998; Deveci & Egginton, 1999) following arteriolar blockade may improve our interpretation of haemodynamic effects by accounting for any change in EDL‐specific perfusion, but no differential effects have previously been reported.

### A cautionary note on peripheral microsphere injections

Bolus injections of saline and washed microspheres did not change FVC, i.e. there was no haemodynamic effect attributable to the administration of microspheres with our protocol. In contrast, administration of suspension medium alone produced a persistent increase in FVC, perhaps due to inclusion of thimerosal (0.01%), an organomercury preservative agent, and the surfactant Tween 80 (0.05%) in the manufacturer's microsphere preparation. Thimerosal induces vascular smooth muscle relaxation and vessel dilation (Forstermann *et al*. 1986; Rosenblum *et al*. 1992), while Tween 80 is associated with a biphasic response whereby arterial blood flow is reduced immediately after injection followed by a hyperaemia (Grund *et al*. 1995). That the microsphere suspension solution causes significant changes in localised blood flow in the rat is of interest due to the widespread use of microspheres to quantify organ‐ and tissue‐specific blood flow (Prinzen & Bassingthwaighte, 2000). Spuriously high rates of blood flow will probably occur near the injection site after administration of diluted microsphere suspensions, confounding haemodynamic outcomes not representative of normal physiological conditions.

### Conclusions

Acute reductions in the number of functional (perfused) capillaries reduced skeletal muscle fatigue resistance, in the absence of changes in blood flow and perfusion pressure. This suggests that peripheral capillary rarefaction *per se* caused a decline in skeletal muscle endurance. Chronic mechanical overload of muscle with a reduced number of functional capillaries successfully recovered fatigue resistance, indicating that the underlying remodelling capacity of muscle can be harnessed to overcome microvascular constraint. A parallel exists between the observations reported here and the increased muscle fatigability observed in patients with CHF, chronic obstructive pulmonary disease and diabetes, supporting the hypothesis that impaired performance of skeletal muscle is in part due to capillary rarefaction, and that the magnitude of rarefaction is a significant aspect of overall muscle performance. These data suggest that significant therapeutic benefits may be accorded to such patients by restoring skeletal muscle microvasculature, by means of flow‐independent angiotherapy to enhance muscle fatigue resistance, ameliorate disease prognosis and improve quality of life.

## Additional information

### Conflict of interest

None declared.

### Author contributions

P.G.T. completed the surgical and experimental work, *P*O_2_ modelling and statistical analyses and drafted the manuscript. P.W.H. completed the histological staining and image analysis. S.E. and H.D. conceived the research, assisted with experimental work and helped draft the manuscript. All authors approved the final version.

### Funding

This work was supported by a British Heart Foundation Project Grant (PG/14/15/30691) to S.E., H.D., K. Witte, D. Hauton and E. A. Gaffney.

## Supporting information


**Statistical Summary Document**
