# Supplementary material for: Impaired skeletal muscle performance as a consequence of random functional capillary rarefaction can be restored with overload‐dependent angiogenesis
Source: J Physiol. 2020 Feb 26;598(6):1187–203. doi: 10.1113/JP278975 (PMC7154729; doi:10.1113/JP278975)
Supplement: Supplementary file 1 — Statistical Summary Document [file TJP-598-1187-s001.docx]

**Manuscript Title: Impaired skeletal muscle performance as a consequence of random functional capillary rarefaction can be restored with overload-dependent angiogenesis**

**Authors:** Peter G Tickle, Paul W Hendrickse, Hans Degens & Stuart Egginton

**Animal model used, if applicable:** Rat: Wistar, Male.

**Underlying hypothesis:** We tested the hypotheses that muscle fatigue resistance is reduced in proportion to a decreased number of functional capillaries, but the capacity for remodelling is retained such that the angiogenic mechanical stimulus of muscle overload can rescue the effects of capillary rarefaction.

**Definitions of ‘n’:**

For all questions ‘n’ refers to the number of animals sampled.

**Statistical summary table:**

| Experimental question number* | Finding/ conclusion | Experimental location/ variable  e.g. cortex vs cerebellum or genotype | Mean value  (or other summary statistic) | SD | n (value) | P** | Units | Data comparisons  e.g. WT vs KO | Statistical test | Any other variable  e.g. subjects’ age or sex | Figure/table in which data are presented | Comments  e.g. observation |
| --- | --- | --- | --- | --- | --- | --- | --- | --- | --- | --- | --- | --- |
| 1: Cardiovascular effects of acute arteriolar occlusion | Microsphere carrying solution caused a transient- localised hyperaemia | Femoral artery | 2 mins: saline 0.011 vs washed MS 0.010 vs MS carrying solution 0.17  5 mins: saline 0.011 vs washed MS 0.010 vs MS carrying solution 0.16 | 2 mins: saline 0.003 vs washed MS 0.004 vs MS carrying solution 0.005  5 mins: saline 0.003 vs washed MS 0.004 vs MS carrying solution 0.004 | 6 | 2 mins: Con vs MS soln. **0.019;** washed MS vs MS soln. **0.001**  5 mins: Con vs MS soln. **0.018;** washed MS vs MS soln. **0.006** | mL min mm Hg-^1^ | Control vs washed microspheres vs carrying solution | ANOVA |  | Results (i) |  |
|  | No bilateral differences in mean resting or end-stimulation femoral flow | Femoral artery | Rest:1.19 vs 1.22  End-stim: 1.71 vs 1.86 | Rest 0.26 vs 0.27  End-stim: 0.39 vs 0.35 | 15 | 0.784 | mL min^-1^ | Control ipsilateral v contralateral limb | ANOVA |  | Results (ii) |  |
|  | No effect of microsphere dose on ipsilateral FVC | Femoral artery | r^2^ = 0.089 |  | 15 | 0.200 | mL min^-1^ mm Hg^-1^ | FVC vs dosage | OLS regression |  | Fig. 1 |  |
|  | Peak (systolic) flow at rest increased with arteriolar occlusion | Femoral artery | r^2^ = 0.210 |  | 15 | **0.019** | mL.min^-1^ | Peak flow vs dosage | OLS regression |  | Results (ii) |  |
|  | Flow amplitude increased with arteriolar occlusion | Femoral artery | r^2^ = 0.156 |  | 15 | **0.045** | mL.min^-1^ | Flow amplitude vs dosage | OLS regression |  | Results (ii) |  |
|  | No effect on arterial blood pressure of increasing arteriolar occlusion | Carotid artery | Before 123; After 122 | Before 10; After 12 | 15 | 0.282 | mm Hg | Before and after microsphere injection | Paired-samples t-test |  | Results (ii) |  |
|  | No effect on pulse pressure of increasing arteriolar occlusion | Carotid artery | r^2^ = 0.009 |  | 15 | 0.661 | mm Hg | Pulse pressure vs dosage | OLS regression |  | Results (ii) |  |
| 2: Chronic effects of arteriolar occlusion/overload on femoral vascular conductance (FVC) | Reduced resting FVC in chronic MS | Femoral artery | Con 8.13; chronic MS 5.37 | Con 1.62; chronic MS 1.12 | 7 | **0.011** | mL min^-1^ mm Hg-^1^ g^-1^ | Con vs chronic MS | ANOVA with Tukey |  | Fig. 2a |  |
|  | No effect on FVC in OV+MS |  | OV+MS 6.38 | OV+MS 2.77 | 8 | 0.132 | mL min^-1^ mm Hg-^1^ g^-1^ | Con vs OV+MS |  |  | Fig. 2a |  |
|  | Reduced resting FVC after OV |  | OV 5.58 | OV 1.47 | 8 | **0.019** | mL min^-1^ mm Hg-^1^ g^-1^ | Con vs OV |  |  | Fig. 2a |  |
| 3: Chronic effects of arteriolar occlusion/overload on magnitude (scope) of functional hyperaemia | No effect on scope in chronic MS | Femoral artery | Con 1.50; chronic MS 1.49 | Con 0.24; chronic MS 0.24 | 7 | 0.999 | n/a | Con vs chronic MS | ANOVA with Tukey |  | Fig. 2b |  |
|  | No effect on scope in OV+MS |  | OV+MS 1.79 | OV+MS 0.31 | 8 | 0.217 | n/a | Con vs OV+MS |  |  | Fig. 2b |  |
|  | Enhanced hyperaemia in OV |  | OV 1.92 | OV 0.51 | 8 | **0.029** | n/a | Con vs OV |  |  | Fig. 2b |  |
| 4: Acute effects on muscle fatigue resistance of microsphere dose/ blood flow restriction | No bilateral difference in FI before microsphere injection | EDL muscle | Ipsi: 46.9 vs contra 48.5 | Ipsi: 8.5 vs contra 8.4 | 25 | 0.493 | n/a | Ipsi. FI vs contralat. FI | ANOVA |  | Results (iv) |  |
|  | Ipsilateral muscle performance was impaired in proportion to microsphere dosage |  | r^2^ = 0.570 |  | 25 | **<0.001** | n/a | Ipsi. FI vs dosage | OLS regression |  | Fig. 3a |  |
|  | Contralateral muscle performance was unaffected by dosage |  | r^2^ = 0.067 |  | 24 | 0.182 | n/a | Contralat. FI vs dosage | OLS regression |  | Fig. 3a |  |
| 5: Chronic effects of arteriolar occlusion/overload on muscle performance | Impaired FI after chronic MS | EDL muscle  EDL muscle | Con 0.47 vs chronic MS 0.36 | Con 0.09 vs chronic MS 0.03 | 7 | **0.016** | n/a | Con vs chronic MS | ANOVA with Tukey  ANOVA with Tukey |  | Fig. 3b; Table 1 |  |
|  |  |  | Chronic MS 0.47 vs OV+MS 0.65 | Chronic MS 0.03 vs OV+MS 0.11 | 8 | **<0.001** | n/a | Chronic MS vs OV + MS |  |  |  |  |
|  | Enhanced FI with OV+MS |  | Con 0.47 vs OV+MS 0.65 | Con 0.09 vs OV+MS 0.11 | 8 | **<0.001** | n/a | Con vs OV+MS |  |  |  |  |
|  | Enhanced FI with OV |  | Con 0.47 vs OV 0.64 | Con 0.09 vs OV 0.08 | 8 | **<0.001** | n/a | Con vs OV |  |  |  |  |
|  | Overload recovers FI in OV+MS |  | OV+MS 0.65 vs OV 0.64 | OV+MS 0.11 vs OV 0.08 | 8 | >0.05 | n/a | OV+MS vs OV |  |  |  |  |
|  | Twitch tension was increased by overload |  | Con 0.35 vs chronic MS 0.35; OV+MS 0.52; OV 0.48 | Con 0.35 vs chronic MS 0.35; OV+MS 0.52; OV 0.48 | Con: 15 Chronic MS: 7 OV+MS: 8 OV:8 | **<0.005** | N | Con vs chronic MS vs OV+MS vs OV |  |  | Table 1 |  |
|  | Mass-specific twitch tension after overload did not differ from control |  | Con 2.41 vs chronic MS 1.90; OV+MS 2.72; OV 2.54 | Con 0.64 vs chronic MS 0.44; OV+MS 0.60; OV 0.40 | Con: 15 Chronic MS: 7 OV+MS: 8 OV:8 | **0.056** | N g^-1^ | Con vs chronic MS vs OV+MS vs OV |  |  | Table 1 |  |
| 6: Acute histological effects of microsphere administration | Reduced ipsilateral capillary perfusion determined FI impairment | EDL muscle | r^2^ = 0.720 |  | 13 | **0.002** | n/a | Ipsi. FI vs perfused capillary index (% total caps. perfused) | Polynomial regression |  | Fig. 4c |  |
|  | Microsphere spill-over to contralateral limb was negligible |  | r^2^ = 0.038 |  | 11 | 0.591 | n/a | Contralat Fi vs perfused capillary index (% total caps. perfused) | OLS regression |  | Results (v) |  |
|  | Capillary domain area increased with dose |  | r^2^ = 0.632 |  | 10 | **0.006** | μm^2^ | CDA vs dose |  |  | Fig. 5a |  |
|  | Heterogeneity of capillary distribution increased with dose |  | r^2^ = 0.487 |  | 10 | **0.025** | n/a | Log RSD vs dose |  |  | Results (v) |  |
| 7: Chronic histological effects of arteriolar occlusion/overload | Overload caused an ipsilateral hypertrophy of the EDL | EDL muscle  EDL muscle | Con 1.02 vs OV 1.19 | Con 0.09 vs OV 0.06 | Con 15 OV 8 | **<0.001** | n/a | Con vs OV | ANOVA with Tukey  ANOVA with Tukey |  | Table 2  Table 2 |  |
|  |  |  | Con 1.02 vs OV+MS 1.19 | Con 0.09 vs OV+MS 0.07 | Con 15 OV+MS 8 | **<0.001** | n/a | Con vs OV+MS |  |  |  |  |
|  | Total capillary density was increased after overload |  | Con 617 vs OV 836 | Con 61 vs OV 131 | Con 11 OV 8 | **<0.001** | mm^2^ | Con vs OV |  |  |  |  |
|  |  |  | OV 836 vs chronic MS 556 | OV 131 vs chronic MS 63 | OV 8 chronic MS 7 | **<0.001** | mm^2^ | OV vs chronic MS |  |  |  |  |
|  | Angiogenic response (increase in cap. density) was not impaired after chronic arteriolar occlusion |  | OV+MS 813 vs OV 836 | OV+MS 114 vs OV 131 | OV+MS 7 OV 8 | 0.969 | mm^2^ | OV+MS vs OV |  |  |  |  |
|  | Functional capillary density was reduced by chronic microsphere administration |  | Con 564 vs chronic MS 420 | Con 107 vs chronic MS 95 | Con 11 chronic MS 7 | **0.049** | mm^2^ | Con vs chronic MS |  |  |  |  |
|  | Functional capillary density was increased by overload |  | Con 564 vs OV 728 | Con 107 vs OV 165 | Con 11 OV 8 | **0.042** | mm^2^ | Con vs OV |  |  |  |  |
|  | Anatomical capillary domain area was increased with chronic arteriolar occlusion |  | Chronic MS 1841 vs Con 1399; OV+MS 1414; OV 1337 | Chronic MS 204 vs Con 156; OV+MS 158; OV 155 |  | **<0.001** | μm^2^ | Chronic MS vs Con/OV+MS/OV |  |  |  |  |
|  | No significant change in chronic perfused capillary domain area with arteriolar occlusion/overload |  | Con 1854 vs chronic MS 2506; OV+MS 2274; OV 1759 | Con 397 vs chronic MS 765; OV+MS 519; OV 521 | Con 11 chronic MS 7 OV+MS 7 OV 8 | >0.05 | μm^2^ | Con vs chronic MS vs OV+MS vs OV |  |  |  |  |
|  | No change in heterogeneity of capillary spacing after chronic arteriolar occlusion/overload |  | Anatomical 0.1023; Perfused 0.1155 | Anatomical 0.010; Perfused 0.015 | Con 11 chronic MS 7 OV+MS 7 OV 8 | Anatomical: 0.139 Perfused: 0.157 | n/a | Con vs chronic MS vs OV+MS vs OV |  |  |  |  |
|  | No change in fibre type composition between chronic groups |  | Type I: 3.64 Type IIa: 21.5 Type IIb/x: 74.9 | Type I: 2.13 Type IIa: 5.21 Type IIb/x: 6.72 | Con 8 chronic MS 7 OV+MS 7 OV 6 | Type I: 0.952 Type IIa: 0.737 Type IIb/x: 0.902 | n/a | Con vs chronic MS vs OV+MS vs OV |  |  |  |  |
|  | Overload caused an increased cross-sectional area of Type IIa fibres |  | Con 933 vs OV+MS 1169; OV 1113 | Con 99 vs OV+MS 120; OV 120 | Con 8 OV+MS 7 OV 6 | OV+MS **0.004;** OV **0.042** | μm^2^ | Con vs OV+MS/OV |  |  |  |  |
|  | Chronic MS and overload caused an increased cross-sectional area of Type IIb/x fibres |  | Con 1644 vs chronic MS 2085; OV+MS 2269; OV 2121 | Con 189 vs chronic MS 310; OV+MS 182; OV 223 | Con 8 chronic MS 7 OV+MS 7 OV 6 | Chronic MS **0.006**; OV+ MS **<0.001**; OV **0.004** | μm^2^ | Con vs chronic MS/ OV+MS/OV |  |  |  |  |
